# Identification and characterization of a novel papillomavirus in thornback skate (Raja clavata)

**DOI:** 10.1099/mgen.0.001541

**Published:** 2025-11-07

**Authors:** Denise da Silva Fong, Joana Abrantes, Teresa Moura, Bárbara Serra-Pereira, Raquel Xavier, Ana Veríssimo, Arvind Varsani, Fabiana Neves

**Affiliations:** 1 CIBIO, Centro de Investigação em Biodiversidade e Recursos Genéticos, InBIO Laboratório Associado, Campus de Vairão, Universidade do Porto, Vairão 4485-661, Portugal; 2 BIOPOLIS Program in Genomics, Biodiversity and Land Planning, CIBIO, Campus de Vairão, Vairão 4485-661, Portugal; 3 Departamento de Biologia, Faculdade de Ciências, Universidade do Porto, Porto 4099-002, Portugal; 4 Division of Modelling and Management of Fisheries Resources, Portuguese Institute for Sea and Atmosphere, Alges, 1495-165, Portugal; 5 CIBIO, Centro de Investigação em Biodiversidade e Recursos Genéticos, InBIO Laboratório Associado, Instituto Superior de Agronomia, Universidade de Lisboa, Lisboa, Portugal; 6 Biodesign Center for Fundamental and Applied Microbiomics, Center for Evolution and Medicine, School of Life Sciences, Arizona State University, Tempe, AZ, USA; 7 Structural Biology Research Unit, Department of Integrative, Biomedical Sciences, University of Cape Town, Observatory, Cape Town 7925, South Africa

**Keywords:** cartilaginous fishes, *Papillomaviridae*, *Secondpapillomavirinae*, thornback skate

## Abstract

Papillomaviruses are non-enveloped, double-stranded DNA viruses capable of infecting a wide range of vertebrates, from chondrichthyans to mammals. In this study, we report for the first time the identification and complete genome of a papillomavirus in the thornback skate (*Raja clavata*), named *Raja clavata* papillomavirus 1 (RclaPV1). The genomic sequence was determined using a metagenomic approach and subsequently confirmed by PCR. The RclaPV1 genome is 5,539 bp in length and displays the typical organization of papillomaviruses, encoding 4 core proteins on a single DNA strand: two early genes (E1 and E2) and two late genes (L1 and L2). Maximum likelihood phylogenetic analyses of the L1 and E1 genes indicate that RclaPV1 belongs to the *Secondpapillomavirinae* subfamily, clustering with fish and amphibian papillomaviruses and showing closer evolutionary relationships to amphibians than to fish.

**Impact Statement**

Through whole-genome sequencing, we determined the first complete genome of a papillomavirus in chondrichthyans that infects the thornback skate (*Raja clavata*). The identification and characterization of *Raja clavata* papillomavirus 1 provide new insights into the evolutionary history of papillomaviruses, particularly those within the *Secondpapillomavirinae* subfamily.

## Data Summary

The authors confirm all supporting data, code and protocols have been provided within the article or through supplementary data files.

## Introduction

Papillomaviruses (PVs) typically cause epithelial and mucosal lesions, such as papillomas and condylomas [1,2]. Papillomaviruses are small, non-enveloped, double-stranded DNA viruses with circular genomes ranging from 5.7 to 8.6 kb in length [3]. These viruses belong to the family *Papillomaviridae*, which is currently divided into two subfamilies: the *Firstpapillomavirinae*, comprising over 50 genera that infect amniotes and the *Secondpapillomavirinae*, which currently only has a single genus – *Alefpapillomavirus*, whose members infect fish and amphibians [4,5]. Fish PVs have relatively smaller genomes, ~5.6 kb in length [3,6], in comparison to mammalian and avian infecting PVs, in which the canine PVs have the largest genomes – 8.6 kb in length [3].

The papillomavirus genome is typically organized into three functional regions and encodes at least four canonical genes, which are among the most conserved across the *Papillomaviridae* family [7]. The early region encodes the E1 and E2 genes, essential for viral replication and transcription. The late region encodes the structural proteins, L1 and L2, which form the viral capsid [8]. Additionally, the genome contains a non-coding regulatory region, the upstream regulatory region (URR) or long control region, between the 5′ end of L1 and the 3′ end of the first early ORF [3,9,11]. Beyond E1 and E2, many PVs encode additional early genes (E4 to E7), with E5, E6 and E7 functioning as viral oncogenes in some human papillomaviruses. These oncoproteins interact and modulate host cellular proteins, contributing to cell transformation and tumour progression. Notably, fish and amphibians’ PVs seem to lack oncogenes, suggesting that the genome of their ancestral PVs contained the minimal backbone (E1, E2, L1 and L2) [7,12].

Chondrichthyans, or cartilaginous fishes, represent the most ancient lineage of extant jawed vertebrates. This group comprises two evolutionary distant subclasses: Elasmobranchii (sharks, rays and skates), and Holocephali (chimaeras), which diverged ~420 million years ago [13]. Despite their basal position in the vertebrate phylogeny, chondrichthyans present a remarkably complex immune system, exhibiting one of the highest diversities of both innate and adaptive components among vertebrates [14,15]. Knowledge about their pathogen communities and viral diseases remains limited, although interest and research in this area have grown in recent years. To date, dsDNA viruses, including polyomaviruses [16,17], herpesviruses [18,19] and adomaviruses [20,21], have been genetically characterized in chondrichthyans. Additionally, papillomas with intranuclear viral particles have been reported in a guitarfish, but genetic characterization has not been achieved [22], and multiple RNA viruses (orthoreovirus, aquareovirus, rotavirus, arterivirus, hepacivirus, calicivirus and flavivirus) have also been detected in elasmobranchs [23].

As part of our ongoing research into the viral diversity associated with chondrichthyans, we sequenced and characterized the complete genome of a novel papillomavirus, *Raja clavata* papillomavirus 1 (RclaPV1), determined from the thornback skate (*R. clavata*), a coastal benthic elasmobranch belonging to the order Rajiformes.

## Methods

### Sampling

Ten individuals of the thornback skate *R. clavata* were caught during the Nephrops Survey Offshore Portugal (NepS (FU 28–29)) and the Portuguese International Bottom Trawl Survey (PT-IBTS), conducted onboard R/V Mário Ruivo in June/July 2022 and October/November 2022, respectively. Both surveys are coordinated by the Portuguese Institute for the Sea and Atmosphere, under the National Biological Sampling Program, as part of the EU/DGMARE Fisheries’ Data Collection Framework. The objective of these surveys is to collect data to support the assessment of the stock status of key species, including Norway lobster (*Nephrops norvegicus*) and deepwater rose shrimp (*Parapenaeus longirostris*), as well as red shrimp (*Aristeus antennatus*) in the case of the Nephrops Survey [24], and hake (*Merluccius merluccius*) and horse mackerel (*Trachurus trachurus*) in the case of the PT-IBTS [25].

### DNA extraction, Illumina sequencing and data processing

DNA was extracted from the spleen of each of the ten thornback skate specimens. Approximately 12 mg of tissue was homogenized in 300 µl of SM buffer (0.1 M NaCl, 50 mM Tris/HCl-pH 7.4, 10 mM MgSO4) and disrupted using a bioruptor (Retsch MM400). The homogenate was centrifuged at 10,000 r.p.m. for 2 min, and 200 µl of the resulting supernatant was used to isolate viral DNA using the High Pure Viral Nucleic Acid Kit (Roche Diagnostic, USA), according to the manufacturer’s instructions. Viral nucleic acid was enriched for circular DNA molecules using rolling circle amplification (RCA) with the TempliPhi™ Kit (GE Healthcare, USA). RCA products were quantified with Qubit™ dsDNA HS Assay kit (Thermo Fisher Scientific, USA), pooled equimolarly and sent to Macrogen Inc. (Korea) for library preparation (Nextera DNA XT) and sequencing on an Illumina Novaseq 6000 platform. The resulting paired-end reads were trimmed using Trimmomatic [26]. In the absence of a reference genome for *R. clavata*, host-derived sequences were removed using Bowtie2 [27] and the reference genome of a close rajiform relative, *Amblyraja radiata* (RefSeq accession no. GCF_010909765.2). The filtered reads were then *de novo* assembled using MEGAHIT v1.2.9 [28].

### Identification of viral genomes

First, the *de novo* assembled contigs were screened for putative viral-like sequences using Diamond [29] against the National Center for Biotechnology Information (NCBI) Reference Sequence (RefSeq) Virus database (version 220) in combination with Cenote-Taker 2 [30]. Contigs longer than 500 nt and with an *e*-value ≤10^−5^ were retained for further analysis. These candidate viral contigs were subsequently analysed using blastx/blastn searches against the NCBI RefSeq_protein and nucleotide (nr/nt) databases, respectively. The papillomavirus identified was used for further analysis.

### Genome amplification and verification

To assess the prevalence of papillomavirus among the ten thornback skate samples, we designed a primer pair targeting the L1 gene (Fwd: 5′ CACTTGCATCAGTGTCCTC 3′: Rev: 5′ CCAATTACAGACGGTGTCATC 3′) based on the sequence of the initially identified PV-like contig. Samples testing positive by PCR were subsequently selected for full genome verification of the virus. For this purpose, multiple overlapping primer pairs (Table 1) were designed using the *de novo* assembled viral genome to amplify the complete viral genome by PCR. Amplification was performed using RCA products as template and the QIAGEN Multiplex PCR Kit (QIAGEN, Hilden, Germany), according to the manufacturer’s instructions. Thermal cycling conditions are detailed in Table 1. Amplicons were resolved on a 2% agarose gel and sequenced using Sanger sequencing. Final sequences were aligned with the PV-like contig using Geneious Prime v2025.0.3 (Dotmatics Inc.).

**Table 1.** Primers and conditions used for PCR amplification and sequencing of RclaPV1 from thornback skate RCA samples

Target gene	Forward primer 5′–3′	Reverse primer 5′–3′	Amplicon size (bp)	PCR condition
*URR/E1*	GCGGAGTCTCTATTTCGCG	GTGTCAGATGGTCCAAG	1,312	95 °C (15′) 40 cycles: 95 °C (45′), 58 °C (20′), 72 °C (1′20′) 60 °C (10′)
*E1*	CTTTGCAATCACACGCTC	GAAGAGTACCACGTGAGTG	1,003	95 °C (15′) 40 cycles: 95 °C (45′), 60 °C (20′), 72 °C (1′20′) 60 °C (10′)
*E2/L2*	CCTGAAGCAGGACTATGC	GGTTGCAGAACAGTCTCG	1,375	95 °C (15′) 40 cycles: 95 °C (45′), 58 °C (20′), 72 °C (1′30′) 60 °C (10′)
*L2/L1*	GTTACAGCTGGAACAGCTG	GGGAGTGTTGAACTGGTAGTTG	1,351	95 °C (15′) 40 cycles: 95 °C (45′), 62 °C (20′), 72 °C (1′30′) 60 °C (10′)
*L1/E1*	CAAGATGGTGACCTGACCTC	GGTGCAAGTGAACTGAGATC	1,343	[touchdown PCR 95 °C (15′) nine cycles: 95 °C (45′), 66–62 °C (20′), 72 °C (1′20′) 31 cycles: 95 °C (45′), 62 °C (20′), 72 °C (1′20′) 60 °C (10′)]

### Viral genome annotation and analysis

ORFs were identified using ORFfinder (https://www.ncbi.nlm.nih.gov/orffinder/) and Geneious Prime v2025.0.3 (minimum length of 25 codons) and confirmed through blast searches. Functional motifs within these genes were detected via manual inspection and the motif search tool (https://www.genome.jp/tools/motif/). Gene annotation was performed in Geneious Prime v2025.0.3. and edited using BioRender (https://www.biorender.com)

To assess sequence similarity, the E1 and L1 genes of the PV-like contig were used to retrieve the closest PV sequences from NCBI and Papillomavirus Episteme (PaVE) (https://pave.niaid.nih.gov/index) databases [31]. A total of nine sequences for the L1 gene and eight for the E1 gene were retrieved. Pairwise aa identities for both genes were calculated using the unweighted pair group method with arithmetic mean as the average linkage method in Sequence Demarcation Tool (SDT) v2 [32].

### Bioinformatic analysis

Since papillomaviruses are classified based on their L1 gene sequence [7], we used this gene to investigate the evolutionary relationships among papillomaviruses. We retrieved all available L1 sequences from the PaVE [31] database, translated them into amino acid sequences and aligned them using MAFFT [33] in Geneious Prime (Dotmatics Inc.). The resulting alignments were trimmed with trimAL [34] to remove poorly aligned regions. A maximum likelihood (ML) phylogenetic tree was then inferred using IQ-TREE v2.4.0 [35] with 1,000 bootstrap replicates, employing the LG+F+I+G4 substitution model as the best-fit under Auto mode. The tree was visualized and annotated using Interactive Tree Of Life (ITOL) v7.1.1 [36].

To generate a more restricted phylogeny focused on the *Secondpapillomavirinae* subfamily, we retrieved the available nucleotide sequences of the *E1*, *E2*, *L1* and *L2* genes from amphibians, teleost fish, birds and testudines, using the PAVE database, and followed a similar pipeline. The best-fit substitution models for each protein alignment were determined using ProtTest v.3.4.2 [37]: LG+G+I for E1, E2 and L1 and VT+G for L2. ML phylogenetic trees were subsequently constructed and visualized as described above. This dataset was also used to perform a comparative genomic analysis of papillomavirus genomes using Clinker [38].

Finally, the individual protein alignments (L1, L2, E1 and E2) were concatenated in Geneious Prime (Dotmatics Inc.), and an ML phylogenetic tree was inferred in IQ-TREE v2.4.0 [35]. The resulting tree was visualized and annotated using iTOL [36].

## Results and discussion

### A novel chondrichthyan papillomavirus

**Fig. 1.**
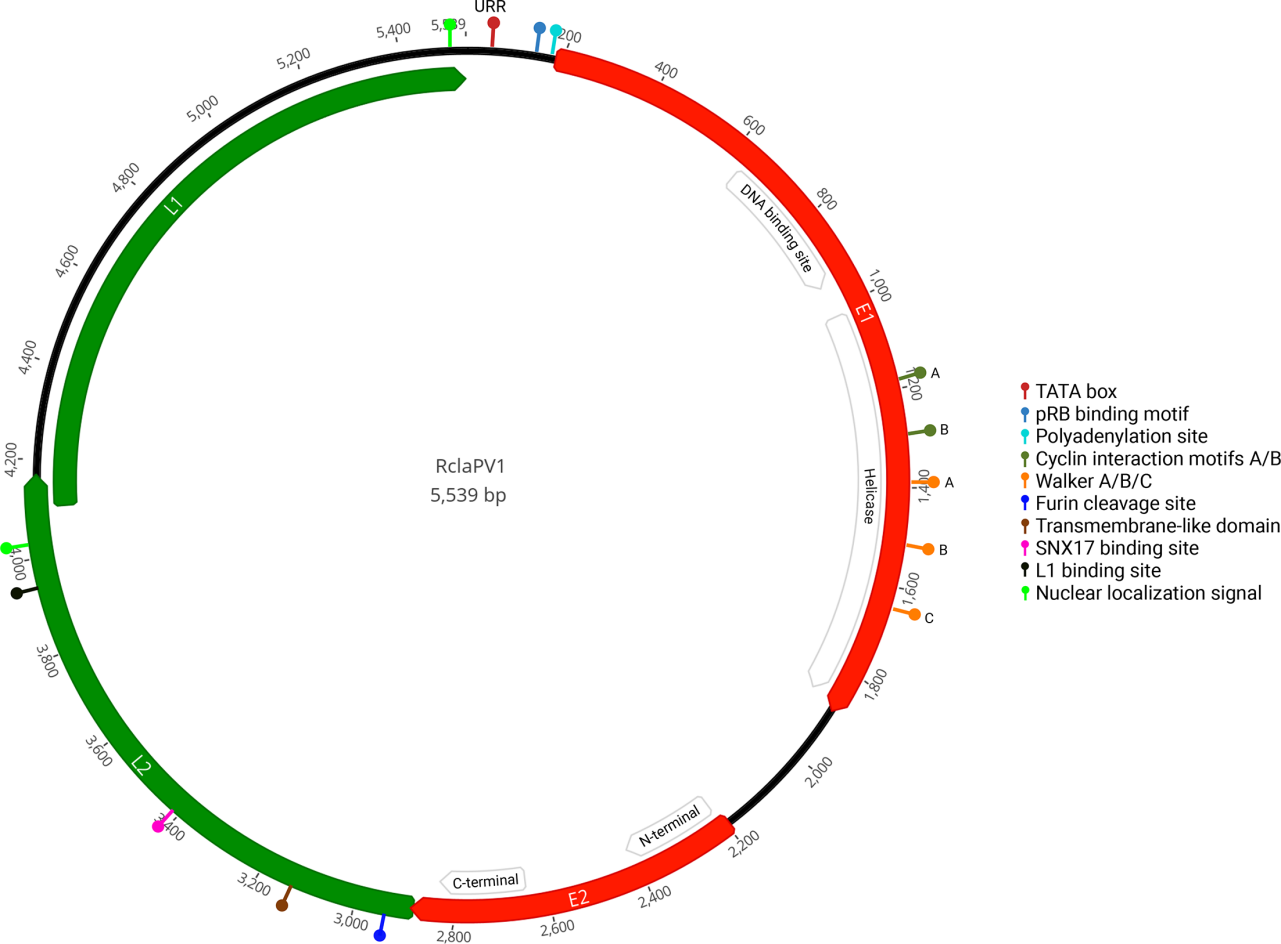
Genomic organization of the thornback skate papillomavirus (RclaPV1) includes the Upstream Regulatory region (URR), and four canonical genes: two early genes (E1 and E2, red) and two late genes (L1 and L2, green). DNA binding site and helicase domains in E1, as well as the N-terminal and C-terminal domains in E2, are indicated by arrowed boxes. Conserved motifs identified are also marked: TATA box (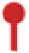), pRB binding motif (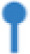) and polyadenylation site (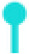) in the URR; two cyclin interaction motifs A/B (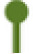), Walker A/B/C (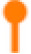) in the E1 protein; and Furin cleavage site (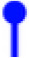), a transmembrane-like domain (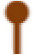), SNX17 binding site (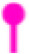), and L1 binding site (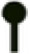) in the L2; and two nuclear localization signals (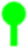) in L2 and L1.

A complete genomic sequence of 5,539 bp was determined from the *de novo* assembly of short-read data and subsequently confirmed by PCR. Among the ten thornback skate specimens analysed, only one tested positive for the presence of the detected papillomavirus. The genome displays the canonical organization observed in papillomaviruses, comprising two early genes (E1 and E2), two overlapping late genes (L1 and L2) and a non-coding upstream regulatory region (URR: 184 bp), all carried in the same strand (Fig. 1), and was named RclaPV1. The *Secondpapillomavirinae* subfamily, which infects fish and amphibians, appears to encompass viral species with relatively small genomes. Although fish papillomaviruses had previously been reported to possess the smallest genomes [4,6], the smallest known PV genome belongs to an amphibian, the Leishan moustache toad (*Leptobrachium leishanense*), with ~5.4 kb [39]. In this context, the 5.5 kb thornback skate PV genome ranks also among the most compact PV genomes identified thus far.

The URR typically resides between the L1 and the first early gene and contains regulatory elements that control early gene expression [40]. In RclaPV1, a URR was identified between L1 and E1 (Fig. 1), containing a putative TATA box, an essential promoter for transcription initiation [41,42]. Although visual inspection was performed to identify motifs such as Nf1 (TTGGC), Sp1 (GGCGGG) [65], AP1 (TGANTCA), Oct1 (AATTGCAT), Tef-1 (TACATACTTC), YY1 (ACATTT) and E2BS (ACC-N6-GGT) [9,41, 43, 44], no additional known transcription factor binding sites were detected. This region does, however, contain a retinoblastoma protein (pRB)-binding motif (LCCTE), typically associated with the oncoprotein E7 and implicated in the disruption of tumour suppressor pathways [6]. It has been hypothesized that the papillomavirus genomic components E6 and E7 seem to have been present in an ancestral genome as an ‘E6/E7-like’ precursor, which gave rise to both genes by duplication [45,46]. Niche adaptation and divergence likely made the encoding proteins evolve new functions tailored to viral requirements, allowing their maintenance in certain genomes [45]. However, throughout evolution, loss of E6 and/or E7 from papillomavirus genomes seems to have occurred several times independently and not associated with particular events [47]. Interestingly, in the papillomavirus uncovered in this study, an evolutionary scar of the E7 protein can still be observed, but not of E6 [48]. A canonical polyadenylation site (AATAAA), important for the processing of late mRNA genes, was also identified in this region [41,42].

The E1 gene encodes a 565 aa (1,698 bp) initiator protein, typically the largest and most conserved protein in papillomavirus genomes. It generally comprises an N-terminal regulatory region, a central DNA-binding domain and a C-terminal helicase domain [49]. In RClaPV1, we identified a DNA-binding motif and a helicase domain containing two putative cyclin interaction motifs (RSL and RWL), which are important for efficient viral replication [50,51]. The characteristic Walker motifs, essential for ATP binding and hydrolysis, were also detected: Walker A (P-loop GPSDTGKT), Walker B (AIDD) and Walker C (LSSN) (Fig. 1).

The E2 gene plays a role in the regulation of viral transcription, replication and genome maintenance. In RclaPV1, it encodes a 230 aa protein (693 bp), representing the smallest E2 among the species analysed. E2 typically includes an N-terminal transactivation domain important to modulate transcription and a C-terminal DNA-binding domain, which were identified in RclaPV1, but other commonly described motifs (e.g. hinge region, DNA-binding domain) could not be detected [44,52, 53].

L1, the major structural capsid protein, lacks highly conserved motifs [54]. However, we were able to identify a putative nuclear localization signal (NLS) in the C-terminal region of the protein. This NLS was detected by visual inspection of the C-terminal region, searching for repeats of arginine (R) and lysine (K) residues, such as KKKRKKKR or RKFKRKTK [44,51]. The L1 protein of RclaPV1 is 478 aa (1,437 bp) in length.

L2, the minor capsid protein, plays a role in facilitating infection and intracellular trafficking [55]. In RclaPV1, the L2 encodes a 429 aa protein (1,290 bp). Several key motifs were identified (Fig. 1), including a furin cleavage site (RRKR), which is essential for infection, a putative transmembrane-like domain (GVTAGTAVGRPG) and an SNX17-binding motif (FRNPAY). In the C-terminal region, we also detected a putative L1 binding site, characterized by multiple proline residues, and an NLS motif composed of lysine-rich sequences [55].

Overall, RclaPV1 displays the minimalistic papillomavirus structure, lacking undisrupted accessory genes typically found in amniotic papillomavirus. This supports the hypothesis that such compact/streamlined genomes may correspond to the genome structure of ancestral proto-papillomavirus [48]. The absence of additional ORFs in PVs infecting basal vertebrates suggests that more complex gene arrangements likely evolved later in the papillomavirus evolution from already existing genomic features, as suggested by the E7 putative signatures in the RclaPV1 genome [45].

### Phylogenetics and pairwise identities

**Fig. 2.**
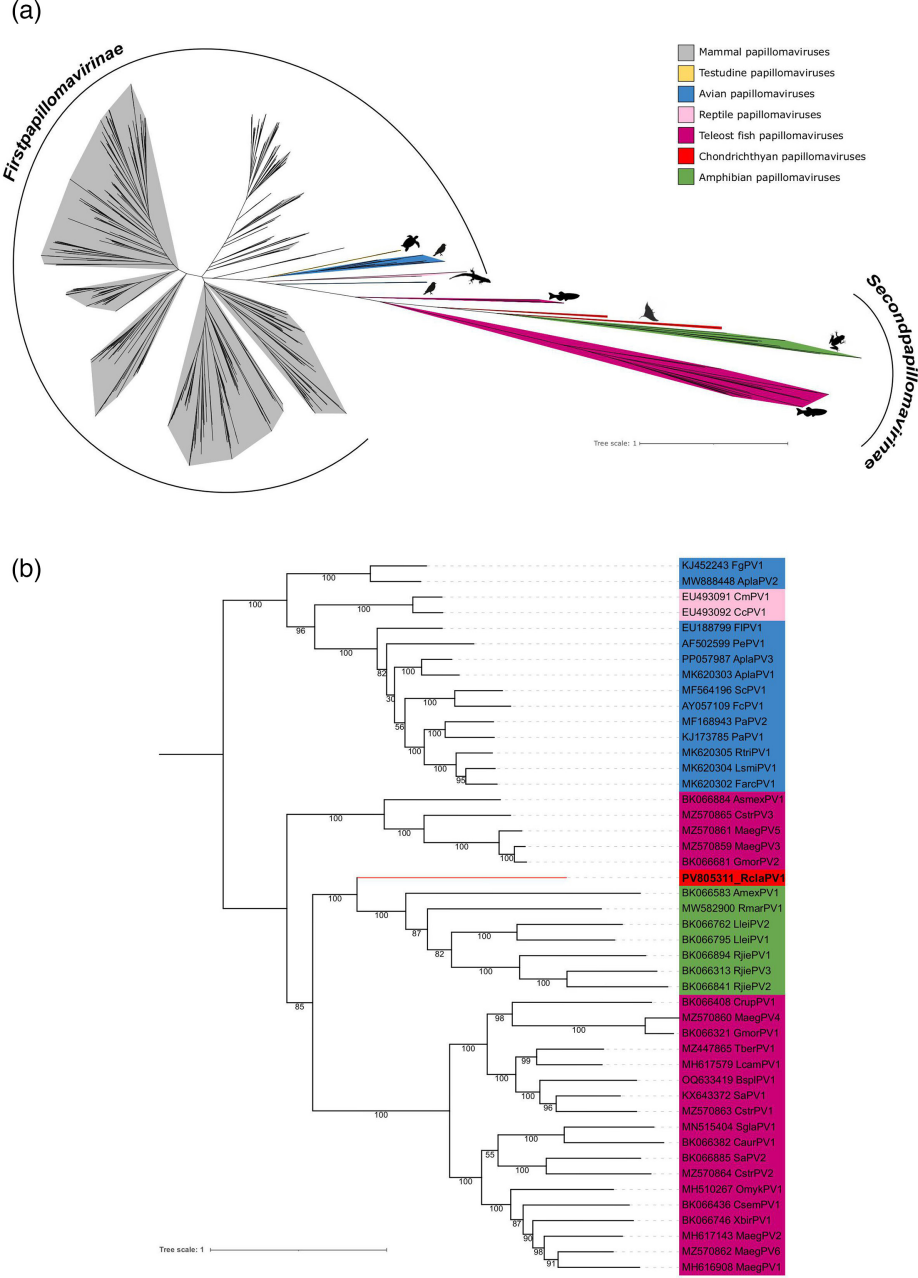
Phylogenetic analysis of the L1 protein. (a) Unrooted maximum likelihood phylogenetic tree with amino acid sequences from all known papillomaviruses, highlighting the *Firstpapillomavirinae* subfamily and the divergent *Secondpapillomavirinae* subfamily. (b) Maximum likelihood phylogeny of concatenated E1, E2, L1 and L2 amino acid sequences of the *Secondpapillomavirinae* subfamily, rooted with the avian and testudine papillomavirus sequences.

Papillomavirus classification is primarily based on the amino acid sequence of the L1 gene [3]. A PV is considered a novel type if its L1 ORF differs by more than 10% from any previously characterized type, while differences between 2 and 10% define subtypes, and those below 2% define variants [7]. Phylogenetic analysis of the L1 gene indicates that RclaPV1 belongs to the *Secondpapillomavirinae* subfamily, being closely related to a papillomavirus of the Sepia stingray (*Urolophus aurantiacus* – UaurPV1) [56] and clustering with amphibian papillomaviruses (Figs 2a and S1A, available in the online Supplementary Material). Our results support the designation of RclaPV1 as a novel papillomavirus representing a new type and species.

Phylogenetic reconstruction based on the protein sequences of L1, L2, E1 and E2 confirmed these phylogenetic affinities, thus confirming the polyphyly of fish papillomaviruses (Fig. 2b). Interestingly, a similar phylogenetic pattern has been previously described in mallard (*Anas platyrhynchos*), where AplaPV2 forms an outgroup relative to AplaPV1 and AplaPV3, separated by two papillomaviruses from testudines [57]. A close phylogenetic affinity between elasmobranch- and amphibian-associated pathogens is not unprecedented, with an unnamed group of Apicomplexa obligate parasites infecting cownose ray (*Rhinoptera bonasus*) and the blue shark (*Prionace glauca*) also showing a close affinity with amphibian and reptile hosts, with no reports of this parasite group in teleost fish so far [58]. Pairwise amino acid comparisons using SDT2 further support the phylogenetic findings. Specifically, RclaPV1 shares 42.18% identity in the L1 protein with UaurPV1, followed by 40.77% identity with the Leishan moustache toad papillomavirus, LleiPV1. For the E1 protein, the highest similarity is to the Jiemuxi brown frog (*Rana jiemuxiensis* – RjiePV3), with 32.53% identity. Lower identities were observed with the fish papillomavirus CaurPV1 (*Carassius auratus* × *Cyprinus carpio*; 26.80%), with the lowest E1 identity found with a papillomavirus retrieved from the roundnose grenadier fish (*Coryphaenoides rupestris* – CruPV1), at 23.23%. It is worth noting that only a full-length L1 sequence is available for UaurPV1, while the E1 sequences from UaurPV1 and L1 from PV2 are partial and, therefore, were not included in these analyses [39].

**Fig. 3.**
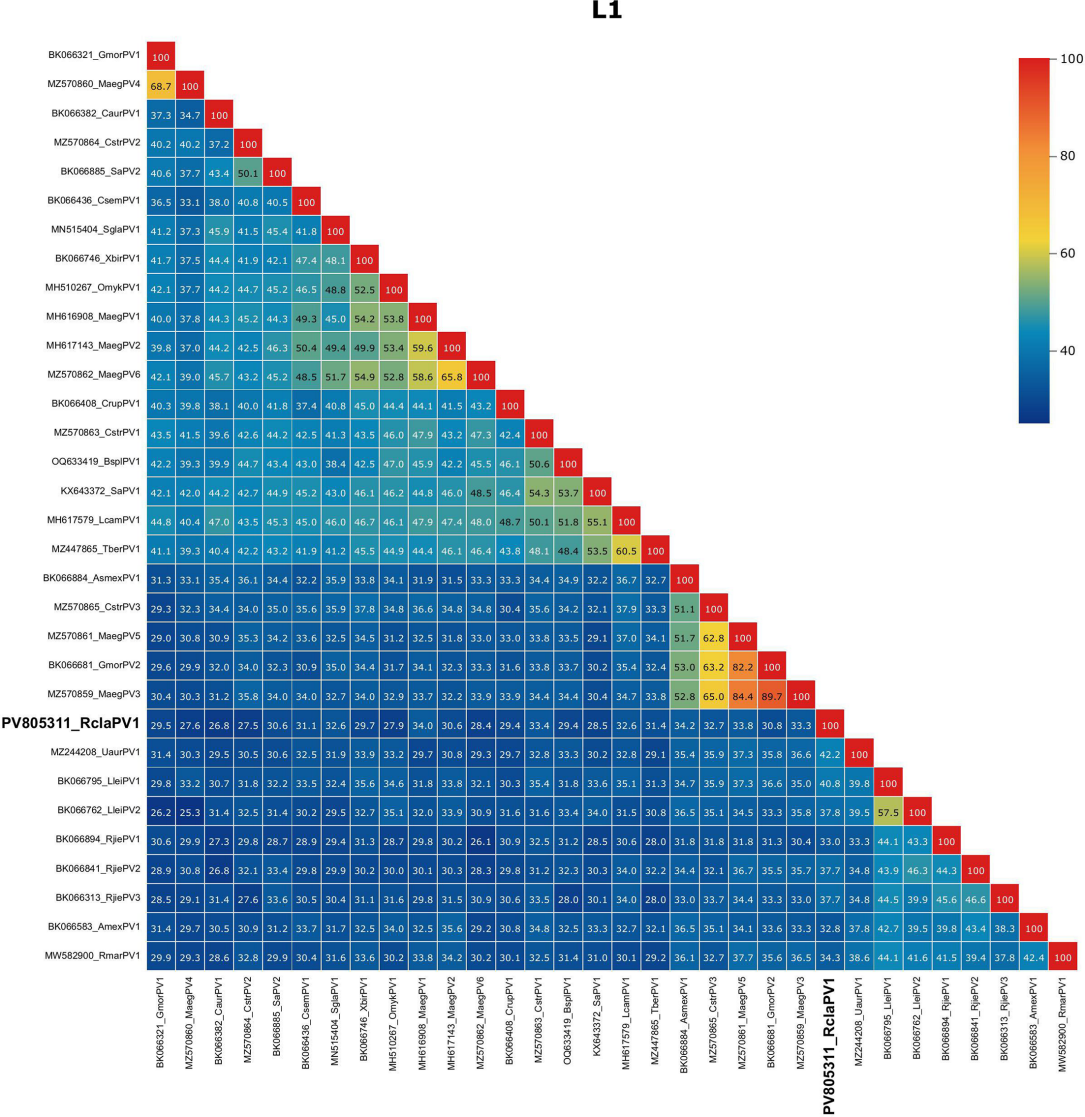
Percentage identity between L1 amino acid sequences of papillomaviruses from the *Secondpapillomavirinae* subfamily. The figure was generated using the SDT2 Virus Classification Tool [32].

Within the *Secondpapillomavirinae* subfamily, the L1 appears to be most conserved with relatively higher percentage pairwise identities to E1, E2 and L2 (Figs 3 and 1B–D). These results are further confirmed using Clinker, where most of the proteins shared low amino acid identity, even between papillomaviruses from the same clade (Fig. S1E).

Remarkably, the data gathered show that the split of the two subfamilies in *Papillomaviridae* is consistent with a dichotomy of terrestrial vs aquatic vertebrates: *Firstpapillomavirinae* comprises PV sequences from terrestrial hosts (reptiles/birds and mammals), while *Secondpapillomavirinae* comprises PV sequences from aquatic hosts (chondrichthyans, teleost fish and amphibians). While there is a signal of host–virus co-speciation in the *Papillomaviridae* phylogeny, there are several cases where viral sequences from a given host do not cluster together [4,59], therefore suggesting other drivers in PV evolution. Ecological speciation has been proposed by King *et al*. [59] to explain the diversification within *Firstpapillomavirinae*; here, we further extend such a proposal to the whole family *Papillomaviriae*.

No signs of infection were observed in any of the collected thornback skate specimens; however, a polyomavirus was previously detected in these samples [17]. In humans, co-infection with both viruses has been studied for their synergistic effect in potentiating oncogenic processes; in fish, co-infections have also been reported, but the biological consequences remain to be determined [3,4, 6]. The discovery of this novel papillomavirus in the thornback skate expands the diversity of the *Secondpapillomavirinae* subfamily and provides novel insights into the evolution of the family *Papillomaviridae*.

## Supplementary material

10.1099/mgen.0.001541 Uncited Fig. S1.

## Abbreviations

ITOL: Interactive Tree Of Life
ML: maximum likelihood
NCBI: National Center for Biotechnology Information
NLS: nuclear localization signal
PaVE: Papillomavirus Episteme
PT-IBTS: Portuguese International Bottom Trawl Survey
PVs: papillomaviruses
RCA: rolling circle amplification
RclaPV1: *Raja clavata* papillomavirus 1
SDT: Sequence Demarcation Tool
URR: upstream regulatory region
