# Supplementary material for: Identification and characterization of a novel papillomavirus in thornback skate (Raja clavata)
Source: Microb Genom. 2025 Nov 7;11(11):001541. doi: 10.1099/mgen.0.001541 (PMC12594251; doi:10.1099/mgen.0.001541)
Supplement: Uncited Fig. S1. [file mgen-11-01541-s001.pdf]

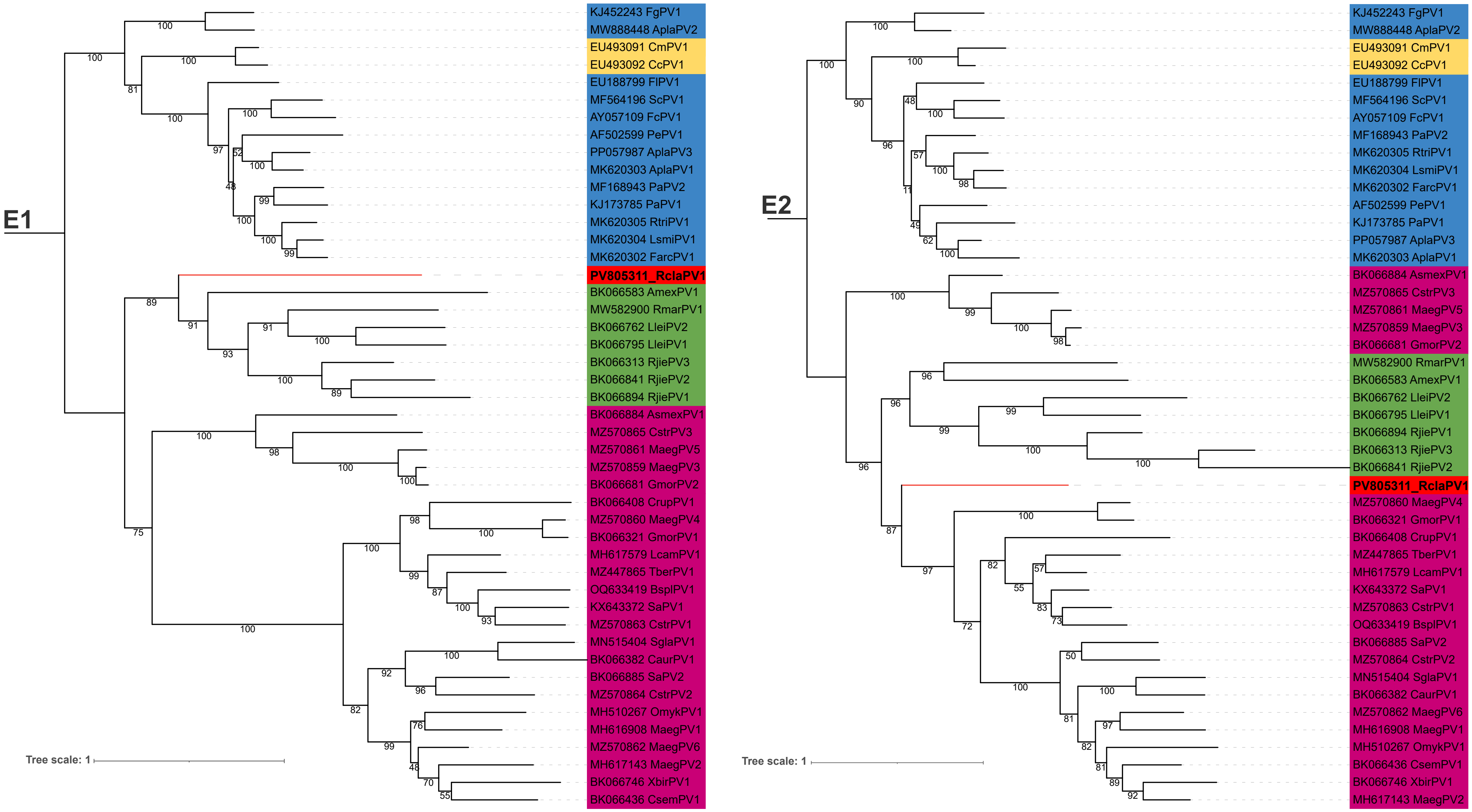

Supplementary Figure 1A. Maximum-likelihood phylogenetic tree of the E1 and E2, L1, and L2 genes for the Secondpapillomavirinae subfamily, rooted with the avian and testudine papillomavirus sequences.

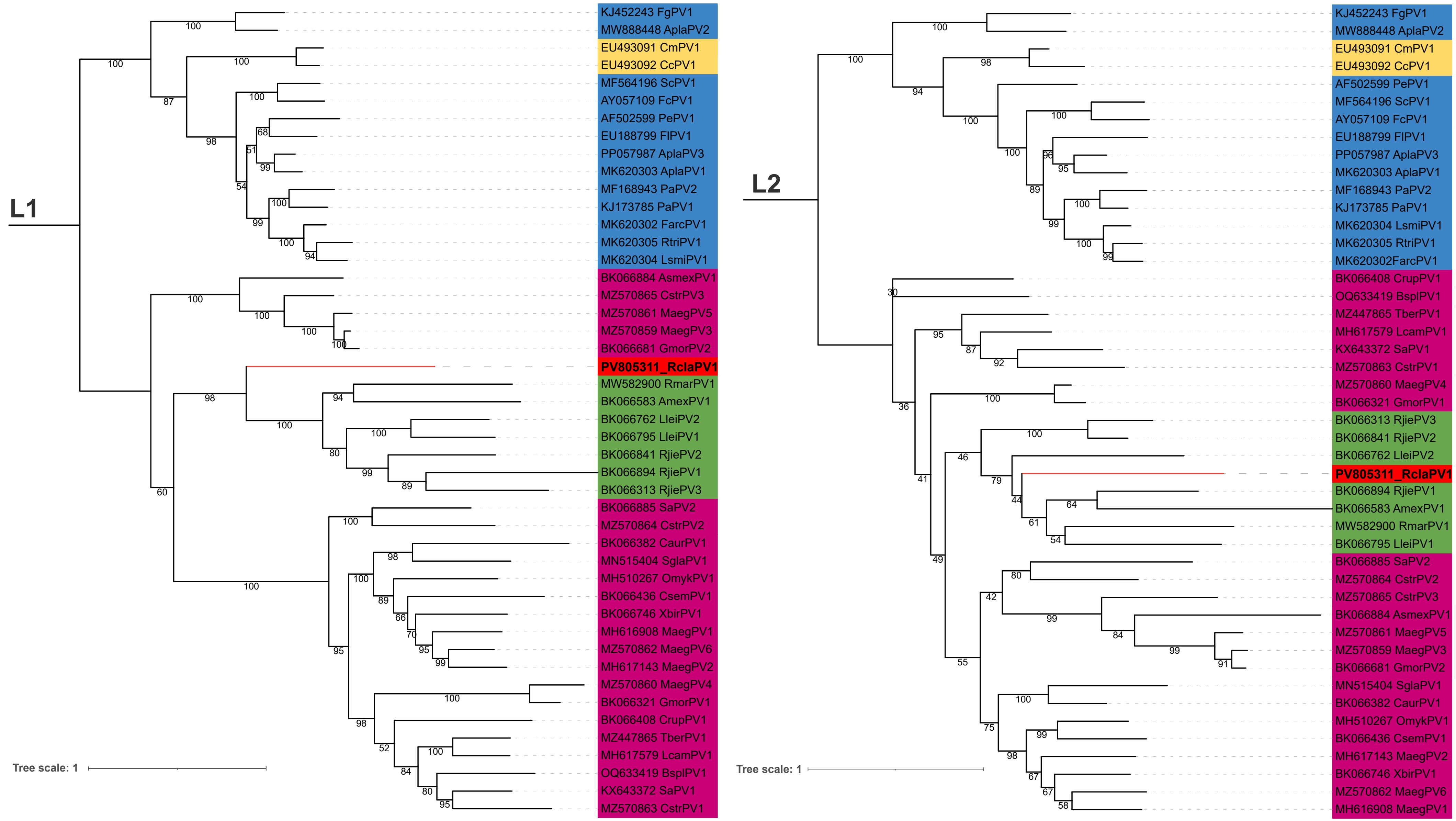

Supplementary Figure 1A (continued). Maximum-likelihood phylogenetic tree of the E1, E2, L1, and L2 genes for the Secondpapillomavirinae subfamily, rooted with the avian and testudine papillomavirus sequences**.**

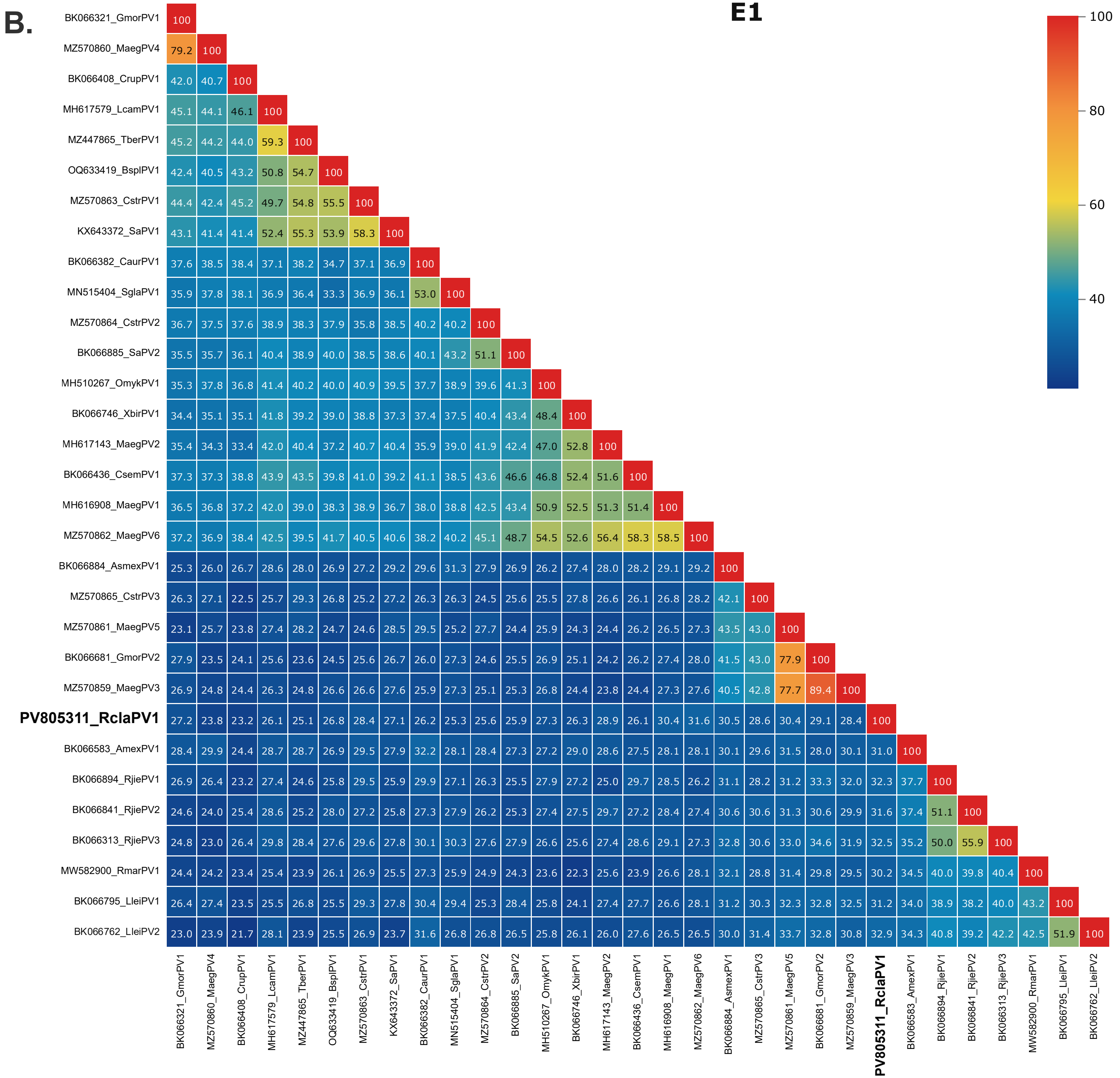

Supplementary Figure 1B. Percentage identity between amino acid sequences of papillomaviruses from the Secondpapillomavirinae subfamily for E1, The figure was generated using SDT2 Virus Classification Tool (1).

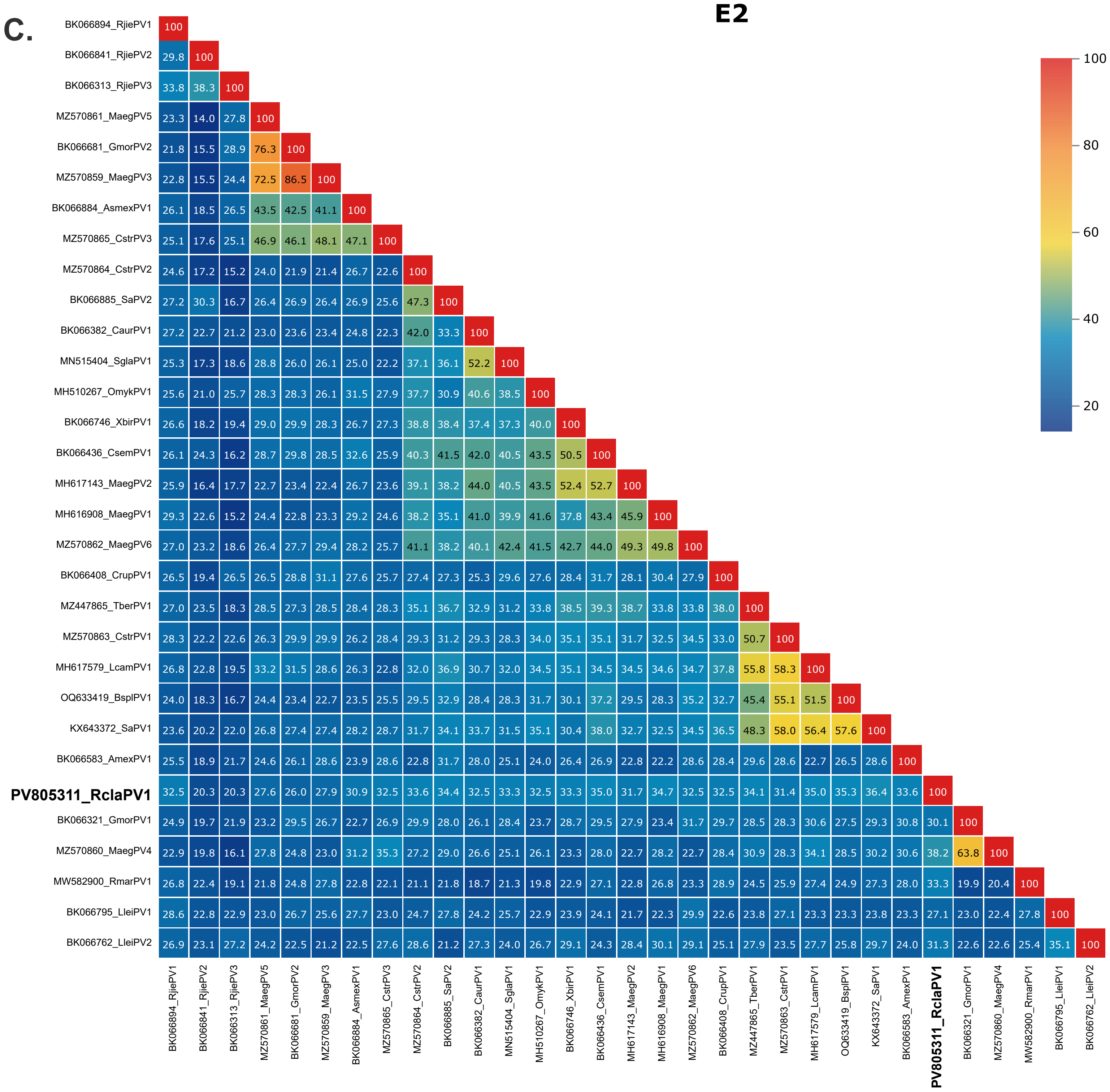

Supplementary Figure 1C. Percentage identity between amino acid sequences of papillomaviruses from the Secondpapillomavirinae subfamily for E2, The figure was generated using SDT2 Virus Classification Tool (1).

D.

L2

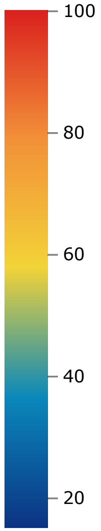

	BK066884_AsmexPV1	MZ570865_CstrPV3	MZ570861_MaegPV5	BK066681_GmorPV2	MZ570859_MaegPV3	BK066382_CaurPV1	MN515404_SglaPV1	BK066436_CsemPV1	MH510267_OmykPV1	MH617143_MaegPV2	MZ570862_MaegPV6	MH616908_MaegPV1	BK066746_XbirPV1	MZ570864_CstrPV2	BK066885_SaPV2	BK066321_GmorPV1	MZ570860_MaegPV4	BK066408_CrupPV1	OQ633419_BsplPV1	MZ570863_CstrPV1	KX643372_SaPV1	MH617579_LcamPV1	MZ447865_TberPV1	BK066583_AmexPV1	**PV805311_RclaPV1**	BK066795_LleiPV1	MW582900_RmarPV1	BK066762_LleiPV2	BK066894_RjiePV1	BK066841_RjiePV2	BK066313_RjiePV3
BK066884_AsmexPV1	100																														
MZ570865_CstrPV3	34.8	100																													
MZ570861_MaegPV5	33.3	38.4	100																												
BK066681_GmorPV2	34.3	40.5	71.3	100																											
MZ570859_MaegPV3	33.5	39.4	71.8	83.4	100																										
BK066382_CaurPV1	28.1	29.7	28.2	28.9	31.0	100																									
MN515404_SglaPV1	24.6	28.0	23.5	23.4	25.1	39.1	100																								
BK066436_CsemPV1	23.1	35.8	29.5	28.6	33.8	36.0	34.9	100																							
MH510267_OmykPV1	21.2	34.0	32.7	33.1	34.8	36.6	35.3	46.9	100																						
MH617143_MaegPV2	22.1	37.3	33.3	25.7	22.9	39.0	35.9	33.7	40.7	100																					
MZ570862_MaegPV6	23.8	26.7	27.2	28.6	27.1	31.4	29.1	40.6	40.0	41.7	100																				
MH616908_MaegPV1	27.8	33.5	24.1	25.9	25.3	39.4	34.5	36.6	39.4	39.1	44.6	100																			
BK066746_XbirPV1	32.0	31.5	29.2	26.2	28.4	43.3	37.5	39.2	40.9	42.4	42.4	50.3	100																		
MZ570864_CstrPV2	22.8	38.8	30.8	27.0	32.6	30.9	27.5	31.8	37.3	34.7	32.2	30.2	37.4	100																	
BK066885_SaPV2	26.3	27.0	26.5	27.8	30.7	31.4	26.9	31.4	32.9	30.3	26.5	29.8	31.5	34.9	100																
BK066321_GmorPV1	23.3	25.1	30.9	26.0	29.1	29.1	27.4	25.1	32.5	31.6	30.5	30.3	25.9	22.1	26.6	100															
MZ570860_MaegPV4	26.4	25.9	28.1	25.9	29.5	28.2	29.7	28.1	31.7	29.9	29.5	30.3	28.1	26.0	27.0	74.0	100														
BK066408_CrupPV1	24.2	28.8	30.0	29.9	27.5	30.9	27.4	31.6	24.1	32.4	24.7	25.9	29.9	25.8	30.4	30.8	29.0	100													
OQ633419_BsplPV1	25.8	31.1	27.6	35.7	29.4	31.4	28.6	31.7	35.5	31.4	30.9	33.5	33.3	30.1	31.7	29.2	28.2	31.9	100												
MZ570863_CstrPV1	27.3	27.0	23.7	23.8	23.8	22.9	25.9	21.7	28.9	22.3	25.9	26.5	25.8	25.3	26.7	28.6	29.6	31.2	32.9	100											
KX643372_SaPV1	26.0	22.4	26.0	26.0	23.6	26.2	26.0	29.8	32.3	33.1	26.4	25.9	27.1	29.6	28.8	28.3	30.6	36.5	35.7	38.3	100										
MH617579_LcamPV1	23.3	30.1	31.7	27.2	27.6	27.6	28.5	26.8	28.7	33.0	29.8	29.2	30.4	27.8	29.5	31.6	33.1	31.2	31.7	40.0	40.0	100									
MZ447865_TberPV1	28.0	31.4	25.2	26.7	31.3	27.6	30.2	26.0	29.6	28.8	27.2	28.6	28.1	29.4	26.5	38.9	38.6	31.7	34.7	38.0	40.1	42.9	100								
BK066583_AmexPV1	20.7	17.7	22.6	19.5	17.8	20.0	25.8	18.9	20.7	23.4	20.0	17.2	22.2	20.2	27.4	18.5	17.2	22.9	21.8	24.4	18.0	19.4	15.4	100							
**PV805311_RclaPV1**	19.3	27.9	25.0	22.4	22.4	24.7	19.1	27.4	28.2	29.7	28.7	23.4	30.1	23.0	24.2	24.6	21.9	21.1	22.2	22.5	22.1	20.7	23.6	24.1	100						
BK066795_LleiPV1	23.8	23.8	26.2	28.6	27.1	30.9	20.2	22.2	26.4	23.3	26.1	25.3	20.9	21.5	27.0	32.5	31.1	28.5	27.3	24.4	24.4	25.7	26.6	25.1	26.9	100					
MW582900_RmarPV1	24.8	28.8	17.7	20.4	24.1	23.9	20.7	25.6	22.6	20.5	26.4	22.1	26.4	27.9	20.5	24.9	23.4	20.4	22.9	24.2	16.9	20.2	23.9	29.8	27.6	35.0	100				
BK066762_LleiPV2	27.4	27.2	32.9	29.0	31.0	28.8	29.1	30.5	26.8	26.6	30.1	26.0	34.7	26.6	27.3	25.8	22.3	27.3	29.4	28.0	26.8	24.4	25.0	22.4	28.5	27.6	28.8	100			
BK066894_RjiePV1	21.3	25.2	24.4	26.2	27.3	28.3	30.1	29.1	25.1	31.3	24.7	23.8	23.8	26.1	23.9	26.5	29.4	25.0	26.9	29.0	21.7	26.1	28.4	26.9	26.7	28.4	28.8	31.3	100		
BK066841_RjiePV2	23.3	26.6	23.3	22.7	23.8	25.7	21.3	22.4	35.5	26.9	24.0	20.0	28.5	25.9	29.0	27.2	25.9	28.8	25.9	24.6	24.3	21.7	23.6	20.2	24.9	26.4	28.1	26.9	29.9	100	
BK066313_RjiePV3	22.2	28.2	24.2	21.8	29.3	29.3	24.6	25.8	25.8	29.3	23.8	28.9	30.4	26.4	24.1	28.2	29.3	25.8	21.7	27.2	22.2	25.1	23.9	30.2	25.8	29.8	31.8	31.0	34.0	55.1	100

Supplementary Figure 1D. Percentage identity between amino acid sequences of papillomaviruses from the Secondpapillomavirinae subfamily for L2, The figure was generated using SDT2 Virus Classification Tool (1).

E.

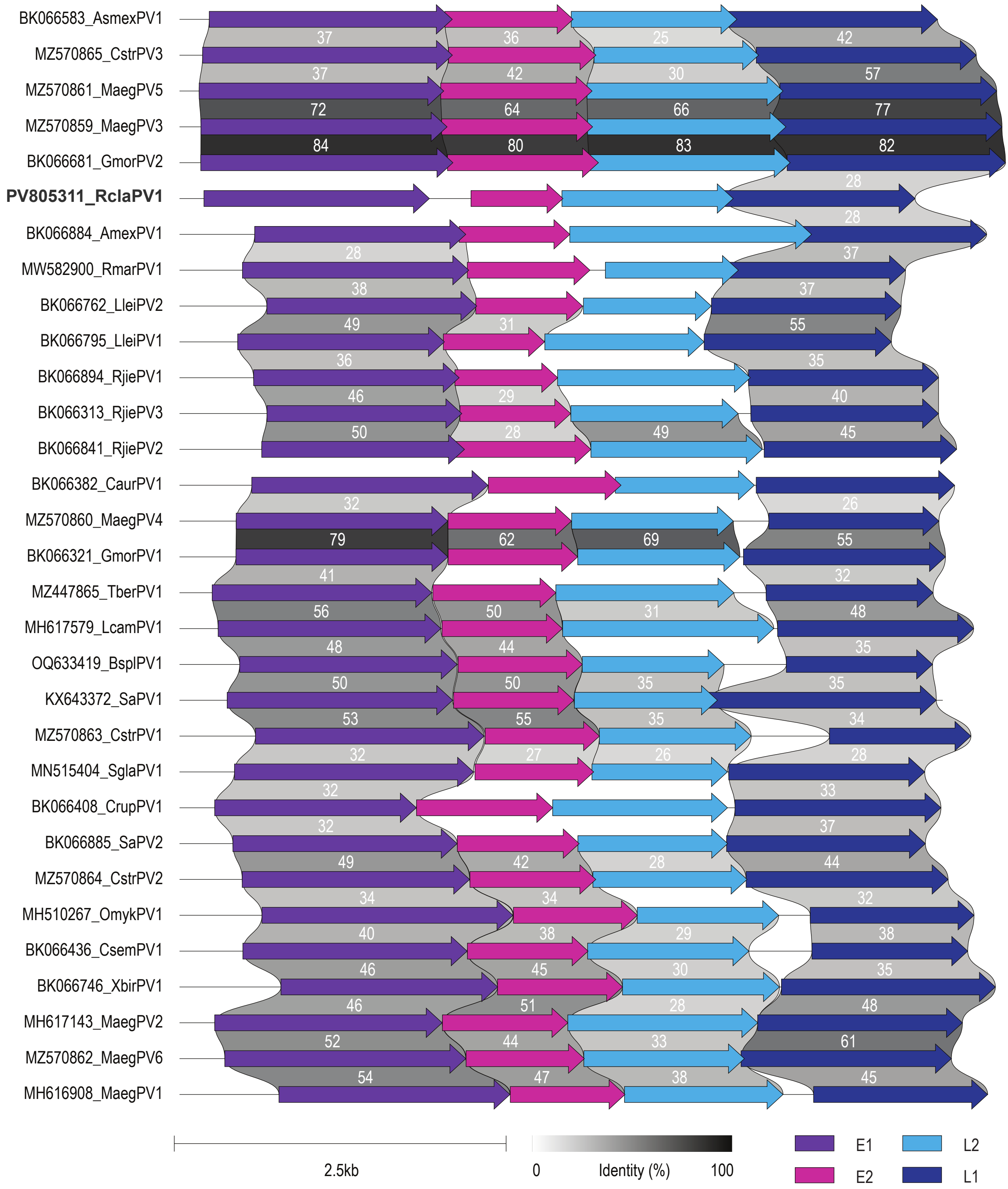

Supplementary Figure 1E. Genome maps based on the protein similarity of papillomaviruses from the *Secondpapillomavirinae* subfamily. Proteins are color-coded (E1 – purple, E2 – pink, L2 – light blue, L1 – dark blue) and amino acid identity is indicated by the grayscale. Maps were drawn with Clinker (2).
